# Low Haemoglobin is Inversely Associated with Disease Activity in Rheumatoid Arthritis: A Cross-Sectional Study from a Tertiary Care Hospital

**DOI:** 10.31138/mjr.210223.lfi

**Published:** 2024-01-25

**Authors:** Sugan Ravichandran, Velammal Petchiappan, Tolstoy Rajangam, Sivakumar Vengudusamy, Vadivelmurugan Nagasubramani Naga Prabu

**Affiliations:** 1 Department of General Medicine;; 2 Department of Rheumatology, PSG Institute of Medical Sciences, Coimbatore, Tamilnadu, India

**Keywords:** rheumatoid arthritis, anaemia, DAS 28, gender difference

## Abstract

**Aim:**

Extra-articular manifestations especially haematological abnormalities are common in Rheumatoid arthritis (RA). The aim is to study the haematological parameters and its correlation with disease severity and gender.

**Methods:**

A cross-sectional study was conducted at a tertiary centre among 50 RA patients who attended the Rheumatology or General Medicine OPD from January 2020 to June 2021. The number of swollen joints, tender joints, the patient’s global assessment, rheumatoid factor, and complete blood counts were recorded. Disease Activity Score (DAS-28) was calculated from these variables and data was analysed using SPSS version 29 with p<0.05 considered as statistically significant.

**Results:**

Of the study subjects, forty (80%) were females; thirty (60%) belonged to the age group 41–50 years. Median age was 42.4(18, 50) years. 79 % (19 out of 24) of subjects with normal haemoglobin had moderate disease activity on DAS 28 score and 50% (13/26) of participants with low haemoglobin levels had high disease activity. Simple linear regression analysis showed low haemoglobin is inversely associated with DAS 28 score (R2 = 0.407, F= 32.888, p < 0.001). Compared to females, males had a higher haemoglobin level which was statistically significant. Female patients had statistically significant higher DAS 28 scores than males (p = 0.016).

**Conclusion:**

Low haemoglobin levels are associated with high disease activity in RA patients and males had less severe disease than females.

## INTRODUCTION

Rheumatoid arthritis (RA) is a chronic, systemic inflammatory condition with an autoimmune pathology.^1^ Apart from the joints, extra-articular involvement of the heart, kidney, lung, digestive system, eye, skin, and nervous system are also quite common.^2^ RA is a condition that is known to affect 5 in every 1000 adults worldwide; its occurrence is 2 to 3 times as common in females as it is in men and can occur at any age with peak incidence observed in the 6th decade of life with a global prevalence of about 1%.^3^ The clinical and histomorphological impression seen in RA is a consequence of inflammation reflected by joint pain, swelling, and eventual destruction of bone and cartilage, alongside systemic manifestations caused due to metabolites of arachidonic acid and different inflammatory cytokines.^4^

Various risk factors have been implicated for RA which includes smoking, obesity, periodontal disease, the gut microbiome, and infections. Multiple factors interact in a genetically susceptible host and studies had shown that HLA-DRB1 is known to be associated with genetic susceptibility and increased disease severity.^5^ Anaemia is quite common in RA both in early as well as long-standing disease; anaemia of chronic disease is the most common cause with peripheral smear showing normochromic normocytic anaemia.^6^ The underlying degree of inflammation associated with RA usually correlates proportionally with the severity of anaemia and serum levels of the acute phase reactants. However, in developing countries such as India, underlying nutritional deficiency associated with debilitating symptoms of RA is often present and is sometimes overlooked.

The present study was conducted to evaluate the prevalence of anaemia in patients with RA and its correlation with the severity of disease activity measured through Disease Activity Score 28 (DAS 28).

## MATERIALS AND METHODS

The present cross-sectional descriptive study was conducted at a tertiary healthcare centre among patients who attended the Rheumatology or General Medicine outpatient Clinic during the study period from January 2020 to June 2021. The study was conducted after obtaining ethical clearance from the Institutional Human Ethics Committee, PSG IMSR, Coimbatore on 20/12/2019 (Project number 19/351)

### Sample size estimation

The sample size was calculated using the formula, n= 2 (1−)/2 1 − ∞/22 is the standard normal rate, (at 5% type 1 error, it is 1.96)

P = Expected proportion in population in India ≈ 3 %
D = absolute error, of 5%

The calculated sample size was 50 patients.

Anaemia was diagnosed as per WHO criteria with a cut-off value of Hb 12g/l or less for women and 13g/dl or less for men.^7^

Inclusion criteria:

1. Patients more than 18 years of age and diagnosed to have Rheumatoid arthritis based on ACR 2010 criteria.
2. Duration of disease less than 10 years of diagnosis

Exclusion criteria:

1. Patients with a previous history of blood transfusion
2. Patients who were diagnosed to have malignancy, chronic kidney disease, DUB, haemorrhoids, or hypothyroidism was excluded from the present study.

The diagnosis of Rheumatoid arthritis was made based on ACR 2010 criteria.^8^ The number of swollen joints and tender joints were noted, and patient global assessment was recorded. The DAS28 score evaluates 28 tender and swollen joint counts, general health (GH; patient assessment of disease activity using a 100 mm visual analogue scale (VAS) with 0 = best, 100 = worst), and levels of an acute phase reactant (either ESR or CRP); Disease activity was classified as mild (2.6 to 3.1), moderate (3.1 to 5.1) and high (>5.1) based on DAS 28 scores.^9^

### Statistical Analysis

The data was analysed using Microsoft Excel and Statistical Package for the Social Sciences (SPSS software Version 29). For all quantitative measurements, mean, median and standard deviation were calculated. Mann Whitney U test and multi-nominal logistic regression analysis were done for analysing statistical differences between the variables and their impact. Determination of the correlation between the severity of anaemia and disease activity was done using Simple linear correlation analysis. A p-value <0.05 was considered statistically significant.

## RESULTS

Of the fifty patients, there were forty females and ten males (F: M ratio of 4:1). The median age of the study population was 42.5 years (18, 50). The median haemoglobin level was 11.85 g% (7.8, 16.2); the majority of them belonged to the age group between 41- 50 years of age **(Table 1)**. The median DAS 28 value was 4.66(2.04, 6.62).

**Table 1.** Demographic details of the study subjects.

**Age group (years)**		
	**Frequency**	**Percentage**
**18–30**	7	14
**31–40**	13	26
**41–50**	30	60
**Gender**		
	**Frequency**	**Percentage**
Male	10	20
Female	40	80

Of the 50 study participants, 31 (62%) had moderate disease activity, 14 (28%) had high disease activity and 5 (10%) had low disease activity. Based on the WHO definition, 26(52%) had anaemia and 24 (48%) had normal haemoglobin levels. Out of the 26 patients with anaemia, 25 were female and there was only one male. Of the 50 study participants, thirty-two (64%) had normal MCV; 38 (76%) showed reduced MCH levels Rheumatoid factor was available for 33 patients; of which twenty–five were reported positive. **(Table 2)**

**Table 2.** Distribution of haemoglobin, DAS 28 score, MCV, MCH, and rheumatoid factor.

**Haemoglobin distribution (n=50)**		
	**Frequency**	**Percentage**
**Low**	26	52
**Normal**	24	48
**DAS 28 score distribution (n=50)**		
**Low activity**	5	10
**Moderate activity**	31	62
**High activity**	14	28
**MCV (n=50)**		
**<80 fl**	18	36
**81–100 fl**	32	64
**MCH (n=50)**		
**<28 pg**	38	76
**>28 pg**	12	24
**Rheumatoid factor (n=33)**		
**Positive**	25	75
**Negative**	8	25

DAS 28 score was tested for association with MCV, MCH, and ESR, the association was shown to be statistically significant for MCV (p=0.005732) and ESR (0.00003454) but not for MCH (p=0.080). **(Table 3)**

**Table 3.** Association of DAS28 scores with MCV, MCH, and ESR.

**DAS 28 score**	**MCV**		
	**<80**	**80–100**	**p value**
**Low activity**	0 (0.0%)	5(100%)	
**Moderate activity**	7(22.6%)	24(77.4%)	0.005732*
**High activity**	9(64.3%)	5(35.7%)	
**MCH**			
	**<28**	**>28**	
**Low activity**	2(40%)	3(60%)	
**Moderate activity**	17(54.8%)	14(45.2%)	0.08031
**High activity**	12(85.7%)	2(14.3%)	
**ESR**			
	**Normal**	**High**	
**Low activity**	3(60%)	2(40%)	
**Moderate activity**	1(3.3%)	30(96.7%)	0.00003454*
**High activity**	0 (0.0%)	14(100%)	

* statistically significant p<0.05

**Figure 1.**
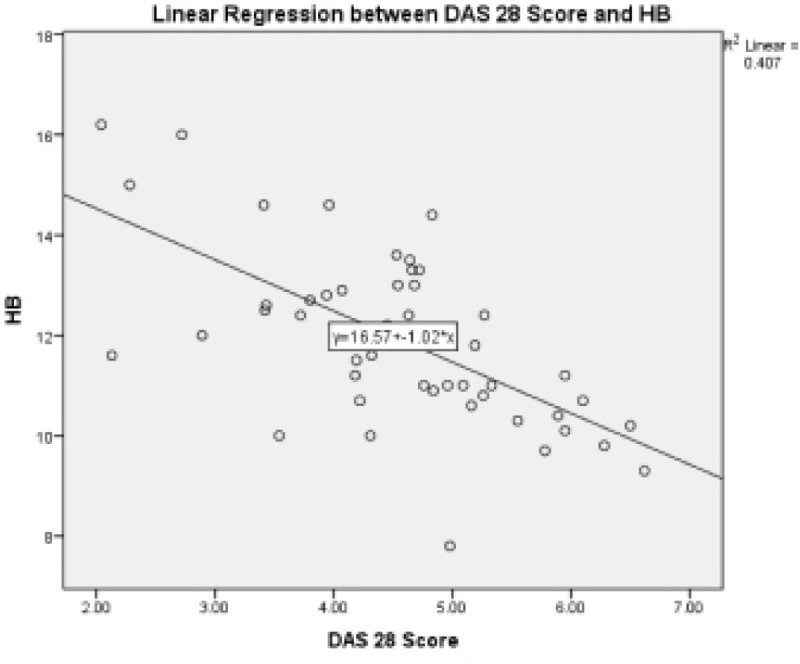
Simple linear regression analysis to test the association of haemoglobin levels with DAS28 scores: showed low haemoglobin levels are associated with high DAS 28 scores.

The majority of the participants (19/24) with normal haemoglobin levels showed moderate activity on DAS 28 score (79%); 50% (13/26) of the participants with low haemoglobin levels showed high activity and this correlation was found to be statistically significant **(Table 4)**. Simple linear regression analysis was used to test the association of haemoglobin levels with DAS28 scores which showed inverse correlation (R2 = 0.407, F= 32.888, p < .001) with lower haemoglobin levels associated with higher DAS28 scores **(Figure 1)**.

**Table 4.** Association of DAS28 score with haemoglobin.

**DAS 28 score**	**Haemoglobin**		**p value**
	**Normal**	**Low**	
**Low activity**	4	1	0.001109 *
**Moderate activity**	19	12	
**High activity**	1	13	

* statistically significant p<0.05

A Mann-Whitney U test was performed to evaluate whether DAS 28 score differed by haemoglobin levels. The results indicated that there was a significant difference between the DAS 28 score of anaemic patients and non-anaemic patients **(Table 5)**. Anaemic patients had significantly higher (Mean rank = 33.19) DAS 28 than non-anaemic patients (Mean rank = 17.17), U = 112.00, p < .001).

**Table 5.** Comparison between patients with low and normal haemoglobin.

**PARAMETERS**	**Low Hb**	**Normal Hb**	**p value**
**Mean age**	40.42(7.63)	41.33(8.45)	0.527
**Female: male**	25:1	5:3	0.0097*
**TJC**	4.69(3.08)	2.58(1.79)	0.010*
**SJC**	5.53(2.59)	3.62(2.63)	0.002*
**DAS28**	5.53(2.59)	3.62(2.63)	0.000*
**HB**	10.61(0.8)	13.3(1.21)	0.000*
**Platelet**	376.11(110.84)	300.2(58.65)	0.006*
**Rheumatoid factor**	119.1(229.9)	114.6(210.8)	0.409
**ESR**	50.03(7.37)	31.75(5.87)	0.001*

Even though male patients were relatively underrepresented in this group we did a sub analysis of the various parameters between male and female rheumatoid arthritis patients. The mean age was similar between the two groups; male patients had a higher haemoglobin level and lower mean DAS 28 score compared to the females which was statistically significant **(Table 6)**.

**Table 6.** Comparison between male and female patients.

	**Male**	**Female**	**p value**
**Mean age**	40.2(7.2)	41.02(2.39)	0.913
**TJC**	2.5(1.47)	3.97(0.9)	0.155
**SJC**	3.8(2.66)	4.82(0.79)	0.050*
**DAS28**	3.77(0.77)	4.76(0.31)	0.016*
**HB**	14.05(1.11)	11.37(0.41)	0.000*
**Platelet**	302.1(49.8)	300.2(58.65)	0.109
**Rheumatoid factor**	35.2(20.6)	139.70(92.21)	0.052
**ESR**	28.2(13.93)	44.5(5.45)	0.079

A Mann-Whitney U test was performed to evaluate whether DAS 28 score differed by gender. The results indicated that there was a significant difference between the DAS 28 score of female patients and male patients. Female patients had significantly higher DAS 28 scores (Mean rank = 27.99) than male patients (Mean rank = 15.55), U = 100.500, p = 0.016). A multinomial logistic regression was performed between the various predictors like age, gender, haemoglobin, platelets, MCV, MCH and Disease Activity in all three groups (Mild, Moderate and Severe disease activity). The traditional 0.05 criterion of statistical significance was employed for all tests. Significant unique contributions were made with age and MCH (p< .001).

## DISCUSSION

RA is a chronic systemic inflammatory auto-immune disease of unknown origin with characteristic persistent symmetric polyarthritis (synovitis) and extra- articular involvement, of which anaemia is a common feature. The aetiology of anaemia in RA is multifactorial which includes anaemia of chronic disease and nutritional deficiency especially iron and folate.10 Multiple reasons have been postulated for this anaemia of chronic disease which include low erythropoietin levels, shortened RBC lifespan, increased levels of inflammatory markers, and reduced erythropoietin response in the bone marrow. The prevalence of anaemia seems to vary between different groups as studies have reported from 30 to 70 %. This may be influenced by the duration of the disease, co-morbid illness and concomitant drug intake especially NSAIDs and the nutritional status of the patients.

In a recent report published in Modern Medicine where 88 patients with Rheumatoid arthritis were analysed, the prevalence of anaemia was noted as 55%, which was similar to our observation (52%)^11^; they noted a higher prevalence (57%) in males than females (50%). However, the prevalence was 83% in females and 5% in males in our study. This might be because of the geographical changes and the nutritional status of the study participants. Also, the mean age of their study population was quite high at 65.31 (12.57) years compared to ours which was 40.86 (7.69) years. While comparing the MCV 64% (32/50) of the study population had normal MCV in our group while in their study it was 81.8%. Also, 36% had low MCV in our group while it was 12.5% in their group.

The present study showed a female preponderance with a prevalence of 80% which is consistent with the observation from other studies.^12,13^ Nikiphorou et al. noted that individuals with anaemia are more likely to be male in their study of haematological abnormalities in new-onset RA.^14^

Our observation was in concordance with the report from a North Indian study with RA of less than 2 years duration where they noted that 67.9% of the study population were anaemic and they too noted that low haemoglobin was associated with higher DAS 28 scores.^15^ However, they also noted that the presence of erosive disease is not influenced by the level of haemoglobin. This is in contrast to the results from the PREMIER trial which showed low haemoglobin is an independent predictor of radiographic joint damage progression.^16^ The radiological correlation was not studied in our patients.

Thrombocytosis is often considered an indirect indicator of inflammation and a study assessing the association between blood platelet, RBC-related indices and disease activity in patients with RA by Xue L et al. concluded that there was a significant association between these indices and RA disease activity which is in concordance with the results of our study.^17^ A post hoc analysis of patients treated with sarilumab conducted by Rubbert Roth A et al. noted that high haemoglobin level is consistently associated with better patient-reported outcomes (PROs) This is consistent with the results of our study with a relationship being shown between haemoglobin levels and disease activity.^18^

Another study by Dechanuwong P et al. evaluated haematological parameters as a predictor of disease remission in RA patients wherein multivariate analysis showed that Hb level, neutrophil-to-lymphocyte ratio (NLR), and mean platelet volume (MPV) were independent factors predicting the disease remission.^19^ Studies conducted by Moller B et al. and Chen Y et al. concluded that Hb levels were significantly related to disease activity and structural damage in RA patients.^16,20^

Irrespective of the gender and nutritional status, what we have noted is that low haemoglobin level is often associated with high disease activity. Consistent across all observations is that more often low haemoglobin is associated with active disease irrespective of the duration of the disease. While gender difference is known to influence the outcome of different aetiologies what is glaring in our observation was that male patients in our group had relatively normal haemoglobin levels and also had lower disease activity which is in contrast to the observations worldwide.^11,14^ One reason might be the small sample size but the majority of the available studies also did have similar sample sizes; the other reason could be dietary factors which may have an indirect effect on haemoglobin and disease activity. The limitations of the study include the single-centred nature of the study and the small sample size. Detailed evaluation for the cause of anaemia and radiological progression was not done. However, this study reinforces the significant association of low haemoglobin with active disease. This underlies an important observation for the clinicians and the correction of anaemia in a better way.

## CONCLUSION

From the results attained, it can be concluded that low haemoglobin levels are associated with high disease activity in RA patients and males had less severe disease than females.

## AUTHOR CONTRIBUTIONS

All authors have read and agreed to the content, and they meet all the four criteria of the International Committee of Medical Journal Editors.
